# *catena*-Poly[[bis­(nitrato-κ*O*)copper(II)]-μ-1,4-bis­(4,5-dihydro-1,3-oxazol-2-yl)­benzene-κ^2^ *N*:*N*′]

**DOI:** 10.1107/S1600536811020605

**Published:** 2011-06-11

**Authors:** Pin-Ning Wang, Chun-Wei Yeh, Hui-An Tsai, Ju-Chun Wang, Maw-Cherng Suen

**Affiliations:** a Department of Materials and Fibers, Graduate School of Materials Applied Technology, Nanya Institute of Technology, Chung-Li 32091, Taiwan; b Department of Chemistry, Chung-Yuan Christian University, Chung-Li 32023, Taiwan; c R&D Center for Membrane Technology, Chung-Yuan Christian University, Chung-Li 32023, Taiwan; d Department of Chemistry, Soochow University, Taipei, Taiwan

## Abstract

In the title coordination polymer, [Cu(NO_3_)_2_(C_12_H_12_N_2_O_2_)]_*n*_, the Cu^II^ ion, situated on an inversion center, is coordinated by two O atoms from two nitrate anions and two N atoms from two 1,4-bis­(4,5-dihydro-1,3-oxazol-2-yl)benzene (*L*) ligands in a distorted square-planar geometry. Each *L* ligand also lies across an inversion center and bridges two Cu^II^ ions, forming a polymeric chain running along the [101] direction. The three O atoms of the nitrate group are disordered over two positions in a 3:2 ratio.

## Related literature

For background to coordination polymers with organic ligands, see: Kitagawa *et al.* (2004 ▶). For related structures, see: Wang *et al.* (2008 ▶). 
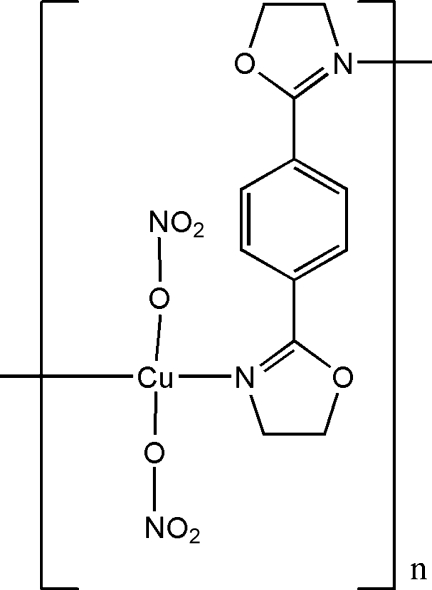


## Experimental

### 

#### Crystal data

[Cu(NO_3_)_2_(C_12_H_12_N_2_O_2_)]
*M* *_r_* = 403.80
Triclinic, 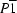

*a* = 6.5240 (8) Å
*b* = 7.5852 (8) Å
*c* = 8.3161 (8) Å
α = 90.393 (2)°
β = 103.556 (2)°
γ = 114.314 (2)°
*V* = 362.09 (7) Å^3^
*Z* = 1
Mo *K*α radiation
μ = 1.56 mm^−1^
*T* = 297 K
0.56 × 0.52 × 0.31 mm

#### Data collection

Bruker SMART 1000 diffractometer
Absorption correction: multi-scan (*SADABS*; Bruker, 1997 ▶) *T*_min_ = 0.433, *T*_max_ = 0.616
2053 measured reflections
1392 independent reflections
1384 reflections with *I* > 2σ(*I*)
*R*_int_ = 0.019

#### Refinement

*R*[*F* ^2^ > 2σ(*F* ^2^)] = 0.034
*wR*(*F* ^2^) = 0.095
*S* = 1.08
1392 reflections
142 parameters
H-atom parameters constrained
Δρ_max_ = 0.29 e Å^−3^
Δρ_min_ = −0.44 e Å^−3^

### 

Data collection: *SMART* (Bruker, 1997 ▶); cell refinement: *SAINT* (Bruker, 1997 ▶); data reduction: *SAINT* and *SHELXTL* (Sheldrick, 2008 ▶); program(s) used to solve structure: *SHELXS97* (Sheldrick, 2008 ▶); program(s) used to refine structure: *SHELXL97* (Sheldrick, 2008 ▶); molecular graphics: *DIAMOND* (Brandenburg, 2009 ▶); software used to prepare material for publication: *SHELXTL*.

## Supplementary Material

Crystal structure: contains datablock(s) I, global. DOI: 10.1107/S1600536811020605/xu5198sup1.cif

Structure factors: contains datablock(s) I. DOI: 10.1107/S1600536811020605/xu5198Isup2.hkl

Additional supplementary materials:  crystallographic information; 3D view; checkCIF report

## Figures and Tables

**Table 1:** Selected bond lengths (Å)

Cu—N1	1.971 (2)
Cu—O2	2.005 (5)
Cu—O3′	1.994 (6)

**Fig. 1.**
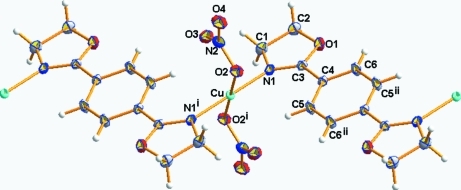
A portion of the chain structure. Ellipsoids are drawn at 30% probability level, and H atoms of spheres of arbitrary radius. Symmetry codes: (i) 1 - x, 1 - y, 1 - z; (ii) -x, 1 - y, -z. The disorder is not shown for clarity.

## supplementary crystallographic information

### Comment

The synthesis of metal coordination polymers has been a subject of intense research due to their interesting structural chemistry and potential applications in gas storage, separation, catalysis, magnetism, luminescence, and drug delivery (Kitagawa *et al.*, 2004). The Ag^I^ complexes containing 1,4-bis(4,5-dihydro-2-oxazolyl)benzene ligands has been reported, which show various two-dimensional networks (Wang *et al.*, 2008). The Cu···Cu distance separated by the bridging ligands is 9.289 (1) Å, while the ligands adopt the *anti* conformation in the structure. The 1-D chain of the title compound forms 3-D supramolecular structure which is interlinked by nitrate anions through C–H···O hydrogen bonds.

### Experimental

An aqueous solution (5.0 ml) of copper nitrate (1.0 mmol) was layered carefully over a methanolic solution (5.0 ml) of 1,4-bis(4,5-dihydro-2-oxazolyl)benzene (1.0 mmol) in a tube. Blue crystals were obtained after several weeks. These were washed with methanol and collected in 55.0% yield.

### Refinement

H atoms were constrained to ideal geometries, with C—H = 0.93 (phenyl) or 0.97 (methylene) Å and *U*_iso_(H) = 1.2*U*_eq_(C). The O atoms of the nitrate group are disordered over two positions in a 3:2 ratio in the structure.

### Crystal data

**Table:**

[Cu(NO_3_)_2_(C_12_H_12_N_2_O_2_)]	*Z* = 1
*M**_r_* = 403.80	*F*(000) = 205
Triclinic, *P*1	*D*_x_ = 1.852 Mg m^−^^3^
Hall symbol: -P 1	Mo *K*α radiation, λ = 0.71073 Å
*a* = 6.5240 (8) Å	Cell parameters from 1976 reflections
*b* = 7.5852 (8) Å	θ = 2.5–26.0°
*c* = 8.3161 (8) Å	µ = 1.56 mm^−^^1^
α = 90.393 (2)°	*T* = 297 K
β = 103.556 (2)°	Parallelepiped, blue
γ = 114.314 (2)°	0.56 × 0.52 × 0.31 mm
*V* = 362.09 (7) Å^3^	

### Data collection

**Table:**

Bruker SMART 1000 diffractometer	1392 independent reflections
Radiation source: fine-focus sealed tube	1384 reflections with *I* > 2σ(*I*)
graphite	*R*_int_ = 0.019
φ and ω scans	θ_max_ = 26.0°, θ_min_ = 2.5°
Absorption correction: multi-scan (*SADABS*; Bruker, 1997)	*h* = −8→7
*T*_min_ = 0.433, *T*_max_ = 0.616	*k* = −8→9
2053 measured reflections	*l* = −7→10

### Refinement

**Table:**

Refinement on *F*^2^	Primary atom site location: structure-invariant direct methods
Least-squares matrix: full	Secondary atom site location: difference Fourier map
*R*[*F*^2^ > 2σ(*F*^2^)] = 0.034	Hydrogen site location: inferred from neighbouring sites
*wR*(*F*^2^) = 0.095	H-atom parameters constrained
*S* = 1.08	*w* = 1/[σ^2^(*F*_o_^2^) + (0.0698*P*)^2^ + 0.1776*P*] where *P* = (*F*_o_^2^ + 2*F*_c_^2^)/3
1392 reflections	(Δ/σ)_max_ = 0.001
142 parameters	Δρ_max_ = 0.29 e Å^−^^3^
0 restraints	Δρ_min_ = −0.44 e Å^−^^3^

### Special details

**Table:**

Geometry. All e.s.d.'s (except the e.s.d. in the dihedral angle between two l.s. planes) are estimated using the full covariance matrix. The cell e.s.d.'s are taken into account individually in the estimation of e.s.d.'s in distances, angles and torsion angles; correlations between e.s.d.'s in cell parameters are only used when they are defined by crystal symmetry. An approximate (isotropic) treatment of cell e.s.d.'s is used for estimating e.s.d.'s involving l.s. planes.
Refinement. Refinement of *F*^2^ against ALL reflections. The weighted *R*-factor *wR* and goodness of fit *S* are based on *F*^2^, conventional *R*-factors *R* are based on *F*, with *F* set to zero for negative *F*^2^. The threshold expression of *F*^2^ > σ(*F*^2^) is used only for calculating *R*-factors(gt) *etc*. and is not relevant to the choice of reflections for refinement. *R*-factors based on *F*^2^ are statistically about twice as large as those based on *F*, and *R*-factors based on ALL data will be even larger.

### Fractional atomic coordinates and isotropic or equivalent isotropic displacement parameters (Å^2^)

**Table:**

	*x*	*y*	*z*	*U*_iso_*/*U*_eq_	Occ. (<1)
Cu	0.5000	0.5000	0.5000	0.02820 (18)	
N1	0.5908 (3)	0.6368 (3)	0.3097 (2)	0.0301 (4)	
N2	0.5360 (5)	0.1919 (3)	0.3813 (3)	0.0423 (5)	
O1	0.6228 (3)	0.7793 (3)	0.0775 (2)	0.0392 (4)	
O2	0.6917 (12)	0.3558 (9)	0.4788 (8)	0.0408 (11)	0.60
O3	0.3378 (13)	0.1675 (8)	0.3452 (9)	0.0520 (14)	0.60
O4	0.6161 (11)	0.0764 (7)	0.3539 (7)	0.0586 (12)	0.60
O2'	0.719 (2)	0.3108 (16)	0.4449 (13)	0.050 (2)	0.40
O3'	0.3542 (17)	0.2391 (10)	0.3667 (12)	0.0364 (15)	0.40
O4'	0.4801 (17)	0.0347 (11)	0.2945 (10)	0.0634 (19)	0.40
C1	0.8447 (4)	0.7532 (4)	0.3336 (4)	0.0412 (6)	
H1A	0.9246	0.6695	0.3378	0.049*	
H1B	0.9119	0.8426	0.4353	0.049*	
C2	0.8593 (4)	0.8615 (4)	0.1836 (4)	0.0423 (6)	
H2A	0.9139	0.9999	0.2149	0.051*	
H2B	0.9643	0.8423	0.1273	0.051*	
C3	0.4874 (4)	0.6623 (3)	0.1666 (3)	0.0282 (4)	
C4	0.2358 (4)	0.5773 (3)	0.0837 (3)	0.0281 (4)	
C5	0.0729 (4)	0.4320 (4)	0.1458 (3)	0.0430 (6)	
H5A	0.1211	0.3858	0.2442	0.052*	
C6	0.1608 (4)	0.6452 (4)	−0.0632 (3)	0.0398 (6)	
H6A	0.2687	0.7432	−0.1060	0.048*	

### Atomic displacement parameters (Å^2^)

**Table:**

	*U*^11^	*U*^22^	*U*^33^	*U*^12^	*U*^13^	*U*^23^
Cu	0.0260 (2)	0.0278 (2)	0.0279 (2)	0.01314 (17)	−0.00148 (15)	0.00318 (15)
N1	0.0231 (9)	0.0304 (10)	0.0319 (10)	0.0115 (8)	−0.0019 (7)	−0.0001 (7)
N2	0.0527 (16)	0.0355 (11)	0.0466 (13)	0.0223 (11)	0.0206 (11)	0.0115 (10)
O1	0.0270 (8)	0.0490 (10)	0.0338 (9)	0.0097 (7)	0.0056 (7)	0.0101 (8)
O2	0.036 (2)	0.038 (3)	0.050 (3)	0.0199 (18)	0.0077 (18)	0.005 (2)
O3	0.042 (3)	0.046 (4)	0.058 (3)	0.016 (3)	−0.0003 (19)	0.000 (3)
O4	0.088 (3)	0.049 (3)	0.062 (3)	0.045 (3)	0.031 (3)	0.013 (2)
O2'	0.042 (4)	0.056 (6)	0.055 (5)	0.028 (4)	0.005 (3)	0.009 (4)
O3'	0.035 (3)	0.025 (4)	0.044 (3)	0.012 (3)	0.000 (2)	−0.001 (3)
O4'	0.102 (6)	0.042 (3)	0.064 (5)	0.044 (4)	0.028 (4)	0.000 (3)
C1	0.0231 (11)	0.0453 (14)	0.0454 (14)	0.0103 (10)	−0.0007 (10)	0.0027 (11)
C2	0.0241 (11)	0.0472 (14)	0.0461 (14)	0.0084 (10)	0.0047 (10)	0.0041 (11)
C3	0.0254 (11)	0.0286 (10)	0.0288 (11)	0.0118 (8)	0.0036 (8)	0.0011 (8)
C4	0.0254 (10)	0.0317 (11)	0.0241 (10)	0.0122 (9)	0.0008 (8)	0.0013 (8)
C5	0.0308 (12)	0.0513 (15)	0.0354 (13)	0.0117 (11)	−0.0018 (10)	0.0199 (11)
C6	0.0272 (12)	0.0457 (14)	0.0354 (13)	0.0074 (10)	0.0028 (9)	0.0170 (11)

### Geometric parameters (Å, °)

**Table:**

Cu—N1^i^	1.971 (2)	O1—C3	1.337 (3)
Cu—N1	1.971 (2)	O1—C2	1.453 (3)
Cu—O2^i^	2.005 (5)	C1—C2	1.498 (4)
Cu—O2	2.005 (5)	C1—H1A	0.9700
Cu—O3'^i^	1.994 (6)	C1—H1B	0.9700
Cu—O3'	1.994 (6)	C2—H2A	0.9700
N1—C3	1.282 (3)	C2—H2B	0.9700
N1—C1	1.484 (3)	C3—C4	1.476 (3)
N2—O2'	1.152 (12)	C4—C5	1.383 (3)
N2—O3	1.190 (8)	C4—C6	1.390 (3)
N2—O4	1.234 (5)	C5—C6^ii^	1.381 (4)
N2—O4'	1.258 (7)	C5—H5A	0.9300
N2—O2	1.340 (7)	C6—C5^ii^	1.381 (4)
N2—O3'	1.354 (10)	C6—H6A	0.9300
			
N1^i^—Cu—N1	180.0	O4—N2—O3'	150.9 (5)
N1^i^—Cu—O3'^i^	92.7 (3)	O4'—N2—O3'	112.3 (6)
N1—Cu—O3'^i^	87.3 (3)	O2—N2—O3'	94.6 (4)
N1^i^—Cu—O3'	87.3 (3)	C3—O1—C2	106.60 (19)
N1—Cu—O3'	92.7 (3)	N2—O2—Cu	103.0 (4)
O3'^i^—Cu—O3'	180.000 (1)	N2—O3'—Cu	103.0 (5)
N1^i^—Cu—O2^i^	88.6 (2)	N1—C1—C2	103.7 (2)
N1—Cu—O2^i^	91.4 (2)	N1—C1—H1A	111.0
O3'^i^—Cu—O2^i^	59.4 (3)	C2—C1—H1A	111.0
O3'—Cu—O2^i^	120.6 (3)	N1—C1—H1B	111.0
N1^i^—Cu—O2	91.4 (2)	C2—C1—H1B	111.0
N1—Cu—O2	88.6 (2)	H1A—C1—H1B	109.0
O3'^i^—Cu—O2	120.6 (3)	O1—C2—C1	104.80 (19)
O3'—Cu—O2	59.4 (3)	O1—C2—H2A	110.8
O2^i^—Cu—O2	180.000 (2)	C1—C2—H2A	110.8
C3—N1—C1	107.4 (2)	O1—C2—H2B	110.8
C3—N1—Cu	137.12 (16)	C1—C2—H2B	110.8
C1—N1—Cu	115.40 (16)	H2A—C2—H2B	108.9
O2'—N2—O3	139.9 (5)	N1—C3—O1	116.8 (2)
O2'—N2—O4	91.9 (5)	N1—C3—C4	128.7 (2)
O3—N2—O4	128.1 (4)	O1—C3—C4	114.5 (2)
O2'—N2—O4'	129.2 (6)	C5—C4—C6	119.0 (2)
O3—N2—O4'	90.1 (6)	C5—C4—C3	122.5 (2)
O4—N2—O4'	39.0 (4)	C6—C4—C3	118.5 (2)
O2'—N2—O2	23.8 (4)	C6^ii^—C5—C4	120.6 (2)
O3—N2—O2	117.1 (4)	C6^ii^—C5—H5A	119.7
O4—N2—O2	114.4 (4)	C4—C5—H5A	119.7
O4'—N2—O2	152.8 (6)	C5^ii^—C6—C4	120.4 (2)
O2'—N2—O3'	117.1 (5)	C5^ii^—C6—H6A	119.8
O3—N2—O3'	22.9 (3)	C4—C6—H6A	119.8

Symmetry codes: (i) −*x*+1, −*y*+1, −*z*+1; (ii) −*x*, −*y*+1, −*z*.
